# Inkjet-Printed Highly Conductive Poly(3,4-ethylenedioxythiophene): Poly(styrenesulfonate) Electrode for Organic Light-Emitting Diodes

**DOI:** 10.3390/mi12080889

**Published:** 2021-07-28

**Authors:** Yadong Liu, Juxuan Xie, Lihui Liu, Kai Fan, Zixuan Zhang, Shiyan Chen, Shufen Chen

**Affiliations:** State Key Laboratory of Organic Electronics and Information Displays, Institute of Advanced Materials (IAM), Nanjing University of Posts & Telecommunications, 9 Wenyuan Road, Nanjing 210023, China; 1219064329@njupt.edu.cn (Y.L.); 1219064217@njupt.edu.cn (J.X.); 1220066016@njupt.edu.cn (K.F.); 1020061836@njupt.edu.cn (Z.Z.); 1220066005@njupt.edu.cn (S.C.)

**Keywords:** organic light-emitting diodes, inkjet printing, PEDOT:PSS, coffee-ring effect

## Abstract

Recently, inkjet printing technology has attracted much attention due to the advantages of drop-on-demand deposition, low-cost and large-area production for organic light-emitting diode (OLED) displays. However, there are still some problems in industrial production and practical application, such as the complexity of ink modulation, high-quality films with homogeneous morphology, and the re-dissolution phenomenon at interfaces. In this work, a printable poly(3,4-ethylenedioxythiophene): poly(styrenesulfonate) (PEDOT:PSS) ink is developed and obtains an adjustable viscosity. Finally, a patterned PEDOT:PSS electrode is fabricated by inkjet printing, and achieves a high conductivity of 1213 S/cm, a transparency of 86.8% and a uniform morphology without coffee-ring effect. Furthermore, the vacuum-evaporated and solution-processed OLEDs are fabricated based on this electrode and demonstrate a current efficiency of 61 cd/A, which is comparable to that of the indium tin oxide counterpart. This work confirms the feasibility of inkjet printing technology to prepare patterned electrodes and expects that it can be used to fabricate highly efficient optoelectronic devices.

## 1. Introduction

In recent years, organic light-emitting diode (OLED) display technology has made great progress in mobile phones, automobiles, household appliances and other fields due to the advantages of self-illumination, wide viewing angle, flexible bending, and lightweight [1,2,3,4,5]. It is well known that indium tin oxide (ITO) is the most commonly used electrode material in OLEDs, which has excellent light transmittance (90%) and high electrical conductivity (10^4^ S/cm) [6]. However, ITO also has some disadvantages, such as the complicated production process (ITO is usually prepared by magnetron sputtering), the high price due to the lack of indium element, and relative brittleness, which is not suitable for flexible wearable devices. Therefore, there are other electrode materials developed for flexible optoelectronic devices, including poly(3,4-ethylenedioxythiophene):poly(styrenesulfonate) (PEDOT:PSS) [7,8], silver nanowires/nanomeshes [9,10], graphene [11], and carbon nanotubes [12]. Compared with the above mentioned electrode materials, PEDOT:PSS has good film-forming ability, high transparency, tunable conductivity, flexibility and solution processability, which can be prepared via spin coating [13], inkjet printing [14,15,16], dip coating [17], spray coating [18], etc. Hence, PEDOT:PSS electrodes have been widely used in flexible optoelectronic devices, such as organic thin film transistors [16], organic solar cells [14,19,20], and OLEDs [7,15,21].

Inkjet printing technology has great potential in high resolution display because of its ability to accurately deposit solutions and achieve patterning [22,23,24]. However, the device performance of optoelectronic devices by inkjet printing technology still lags far behind the evaporated devices, due to that the smooth and uniform film morphology is a big challenge for inkjet printing. The film morphology is determined by many factors, such as the ink viscosity, the ink surface tension, the boiling point of the solvents, and the ink spreadability on different substrates [25,26,27,28,29]. For instance, Teo et al. used a microreactive inkjet printing (MRIJP) method to pattern various 2-dimensional (2D) and 3D structures of PEDOT:PSS/ionic liquid (IL) hydrogel through in-air collision and coalescence. The patterned PEDOT:PSS films prepared by the MRIJP method are high conductive (900 S/cm) and transmittance (89% in the visible range) [30]. An alternating current-electroluminescent device with ZnS:Cu phosphor mixed in poly(dimethyl siloxane) matrix was fabricated using this PEDOT:PSS/IL as the top and bottom electrodes. Mu et al. used a varied droplet-spacing method to improve the film uniformity of PEDOT:PSS on ITO, which was formulated by diluting PEDOT:PSS with water and ethylene glycol (EG) [31]. However, this PEDOT:PSS film had a low conductivity and was used as a hole injection layer in the final OLEDs based on the ITO anode. Deferme et al. reported a five-layer PEDOT:PSS electrode with a sheet resistance of 45 Ω/sq and a transparency of 95% [32]. Nevertheless, the roughness of the film was as high as 35 nm, which was not suitable for optoelectronic devices.

In order to improve the viscosity and surface tension of the PEDOT:PSS ink, Lee et al. proposed to use glycerol and surfactant. Then, the PEDOT:PSS film with a conductivity of 162 S/cm was obtained, and a polymer solar cell with a 3.16% power convert efficiency was fabricated by inkjet printing technology [33]. Yoon et al. added dimethyl sulfoxide (DMSO) and fluorinated surfactant (Zonyl FS-300) into PEDOT:PSS ink for proper viscosity and surface spreadability, and then printed DMSO solvent on the prepared PEDOT:PSS film to further improve the electrical conductivity. Through the double-shot inkjet printing, the conductivity of PEDOT:PSS film reached higher than 1000 S/cm [8]. Eventually, an OLED based on the printed PEDOT:PSS electrode was fabricated with a luminance of 1863 cd/m^2^. It is worth noting that the device performance of the optoelectronic devices based on the inkjet-printed PEDOT:PSS electrode is inferior to that of the devices with ITO electrode, which can be attributed to the higher surface roughness and the lower conductivity of PEDOT:PSS electrode compared with ITO.

In this paper, we enhanced the conductivity and spreadability of PEDOT:PSS ink on the rigid and flexible substrates by adding DMSO and Triton X-100. Furthermore, in order to achieve the printable PEDOT:PSS ink, EG and deionized water (DI water) were used to adjust the surface tension and viscosity. Thus, this inkjet-printed PEDOT:PSS film had a sheet resistance of 97 Ω/sq (the conductivity is 1213 S/cm) and a transmittance of 86.8% at 550 nm. Meanwhile, the sheet resistance of this PEDOT:PSS electrode printed on the flexible polyethylene terephthalate (PET) substrate can remain 75% of the original value after bending 600 times, indicating the good mechanical flexibility. Finally, the current efficiency of the OLED based on this inkjet-printed PEDOT:PSS electrode reached 61 cd/A, which was comparable to that of the ITO device. This work demonstrates the feasibility of inkjet printing technology in high performance optoelectronic devices.

## 2. Materials and Methods

### 2.1. Materials

The high-conductive PEDOT:PSS (CLEVIOS PH 1000) and hole-injection PEDOT:PSS (CLEVIOS P VP AI 4083) were purchased from Heraeus (Hanau, Germany). DMSO, chlorobenzene (CB), Triton X-100, and EG in the ink formulation were bought from Sigma-Aldrich (Saint Louis, MO, United States). OLED materials were purchased from Han Feng Chemical (Shanghai, China), such as TAPC (1,10-bis(di-4-tolylaminophenyl) cyclohexane), PVK (poly(*N*-vinyl carbazole)), mCP (1,3-bis(carbazol-9-yl)benzene), TCTA (tris(4-carbazoyl-9-ylphenyl)amine), 26DCzPPy (2,6-bis[3 -(carbazol-9-yl)phenyl]pyridine), Ir(ppy)_3_ (tris(2-phenylpyridine) iridium), Ir(ppy)_2_(acac) (Bis(2-phenylpyridinato-C2,N) (acetylacetonate)iridium(III)), and TmPyPB (1,3,5-tri[(3-pyridyl)-phen-3-yl]benzene). LiF was purchased from Vizu Chemical (Shanghai, China). Al was purchased from Zhongnuo Company (Beijing, China).

### 2.2. Preparation of High Conductive PEDOT:PSS Ink and Light-Emitting Solution

Firstly, 5 vol.% DMSO and 0.05 vol.% Triton X-100 were added to the pristine PEDOT:PSS solution, named m-PEDOT:PSS ink. In addition, 40 vol% EG and 20 vol% DI water were added to dilute the modified ink and stirred for 3 h. The solution of the light-emitting layer was prepared by blending PVK:TCTA:26DCzPPy:Ir(ppy)_2_(acac) × (10 wt.%:40 wt.%:40 wt.%:10 wt.%) in CB at a concentration of 20 mg/mL.

### 2.3. Inkjet Printing and Characterization of the PEDOT:PSS Film

Firstly, the glass and PET substrates were ultrasonically treated in ethanol, acetone, and deionized water for 15 min each time. Then, the substrates were UV treated in ozone for 30 min. Next, m-PEDOT:PSS ink with EG and DI water was filtered from a 0.45 μm PTFE filter before use. A Fujifilm Dimatix printer (DMP-2850, Santa Clara, CA, United States) was used for inkjet-printing process in this work. During the inkjet-printing process, the nozzle voltage was set at 25 V, the drop spacing was 30 μm, the printing height was 0.5 mm, and the driving waveform was depicted in the following discussion. Finally, the inkjet-printed PEDOT:PSS film was baked at 60 °C and 120 °C for 15 min, successively.

The ink viscosity was measured by a rotational viscometer (Brookfield DVESLVTJO, Middleboro, MA, United States). The thicknesses of the PEDOT:PSS films were obtained from a surface profilometer (Bruker DektakXT, Billerica, MA, United States). The sheet resistance of the film was characterized by a 4-point probes resistivity measurement system (RTS-9, Guangzhou, China). The transmittance of the film was measured by an ultraviolet−visible absorption spectra instrument (PerkinElmer Lambda 35 S, Waltham, MA, United States). The film morphology was characterized by optical microscope (OM, Zeiss Primotech MAT cod., Heidenheim, Germany) and atomic force microscopy (AFM, Bruker Dimension ICON, Billerica, MA, United States). The work function of the m-PEDOT:PSS electrode, PEDOT:PSS HIL, and ITO were characterized by ultraviolet photoelectron spectroscopy (UPS, Kratos Axis Supra, Manchester, UK).

### 2.4. Fabrication and Characterization of OLEDs

The vacuum-evaporated OLED structure was PEDOT:PSS electrode/PEDOT:PSS hole injection layer (HIL)/TAPC (20 nm)/mCP:Ir(ppy)_3_ (20 nm)/TmPyPB (45 nm)/LiF (0.5 nm)/Al (100 nm). The PEDOT:PSS (CLEVIOS P VP AI 4083) solution was deposited by spin-coating at 3000 rpm for 60 s on a pre-printed PEDOT:PSS anode, and then annealed at 120 °C for 30 min, named the PEDOT:PSS HIL. Then, TAPC/mCP:Ir(ppy)_3_/TmPyPB/ LiF/Al were successively deposited by vacuum evaporation under 6 × 10^−4^ Pa. Additionally, the solution-processed OLEDs were fabricated according to the device structure of PEDOT:PSS electrode/PEDOT:PSS HIL/PVK:TCTA:26DCzPPy:Ir(ppy)_2_(acac) (30 nm)/mPyPB (50 nm)/LiF (0.5 nm)/Al (100 nm). The PVK:TCTA:26DCzPPy:Ir(ppy)_2_(acac) layer was spin-coated at 3000 rpm for 60 s and annealed at 80 °C for 30 min in a glove box. Eventually, TmPyPB/LiF/Al were successively deposited by vacuum evaporation under 6 × 10^−4^ Pa. In the end, the current density (*J*)—voltage (*V*)—luminance (*L*) characteristics, and electroluminescence (EL) spectra were measured by a source meter (Keithley 2400, Mansfield, TX, United States) and a spectroradiometer (PR-655 SpectraScan, NK, United States).

## 3. Results and Discussion

**Figure 1:**
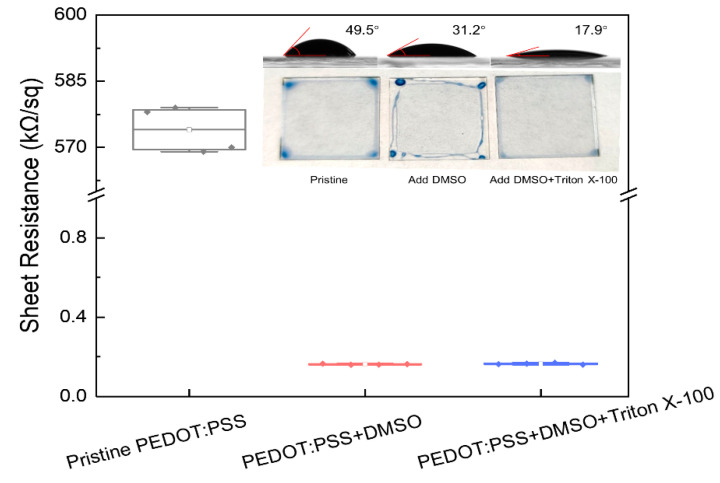
The sheet resistance of PEDOT:PSS films with different ink formulations (inset: the contact angle and film image of pristine PEDOT:PSS, PEDOT:PSS added DMSO, and PEDOT:PSS added DMSO and Triton X-100 on the glass substrates, respectively).

In order to fabricate a PEDOT:PSS electrode with superior conductive performance, the ink was firstly modified by adding DMSO and Triton X-100 to improve its electrical conductivity and the spreading ability, respectively. As shown in Figure 1, with the addition of DMSO, the sheet resistance of the PEDOT:PSS film decreased from 570 to 0.16 kΩ/sq. However, the DMSO modified PEDOT:PSS film was not homogeneous due to the high viscosity (Table 1). As the contact angle shown in the illustration of Figure 1, when DMSO was added to the pristine PEDOT:PSS ink, the contact angle decreased from 49.5° to 31.2°, because the ink was slightly diluted. In order to further improve the spreadability, the surfactant Triton X-100 was added to the ink, and the contact angle decreased from 31.2° to 17.9°. The utilization of Triton X-100 significantly improved the spreading property of the ink. Moreover, the sheet resistance was not influenced by using 0.05 vol.% Triton X-100. It can be concluded that the addition of DMSO can improve the conductivity, and the utilization of Triton X-100 can improve the spreadability of PEDOT:PSS ink [34]. The high conductive PEDOT:PSS ink modified by DMSO and Triton X-100 was named m-PEDOT:PSS ink in the following discussion.

**Figure 2:**
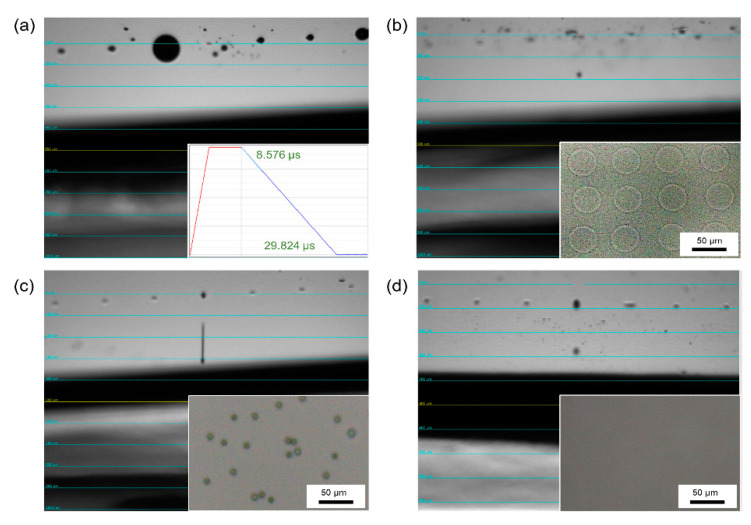
Jetting behaviors of (**a**) the m-PEDOT:PSS ink (Inset: The waveform of DI water), (**b**) the m-PEDOT:PSS ink with DI water, (**c**) the m-PEDOT:PSS ink with EG, and (**d**) the m-PEDOT:PSS ink with EG and DI water (Inset: the optical microscopy images of inkjet-printed dots).

The viscosity of m-PEDOT:PSS ink was firstly measured, as shown in Table 1. The viscosity of the m-PEDOT:PSS ink was 44 cP. As shown in Figure 2a, the nozzle was blocked due to the high viscosity. Thus, the m-PEDOT:PSS ink was diluted with DI water, and the viscosity was decreased to 17 cP. The ink was jetted normally and printed to a regular dot matrix as shown in Figure 2b. Nevertheless, it was found that there was coffee-ring effect in the printed dots as shown in the optical microscope images. It was reported that the combination of high- and low-boiling point solvents can promote the Marangoni flow phenomenon, which moves solutes from the ends of the droplet to the middle and optimizes the film formation properties [31]. Therefore, we added EG with the boiling point of 197 °C to eliminate the coffee-ring phenomenon of the m-PEDOT:PSS ink. As shown in Figure 2c, the m-PEDOT:PSS ink with EG was successfully jetted out. However, the ink appeared to be trailing and had satellite droplets due to the relatively high viscosity of 25 cP. Furthermore, DI water was utilized to decrease the viscosity of EG added m-PEDOT:PSS ink. Finally, the m-PEDOT:PSS ink added EG and DI water obtained a viscosity of 11 cP, which can be jetted well as shown in Figure 2d. Moreover, the printed dots was homogeneous without the coffee-ring effect.

**Figure 3:**
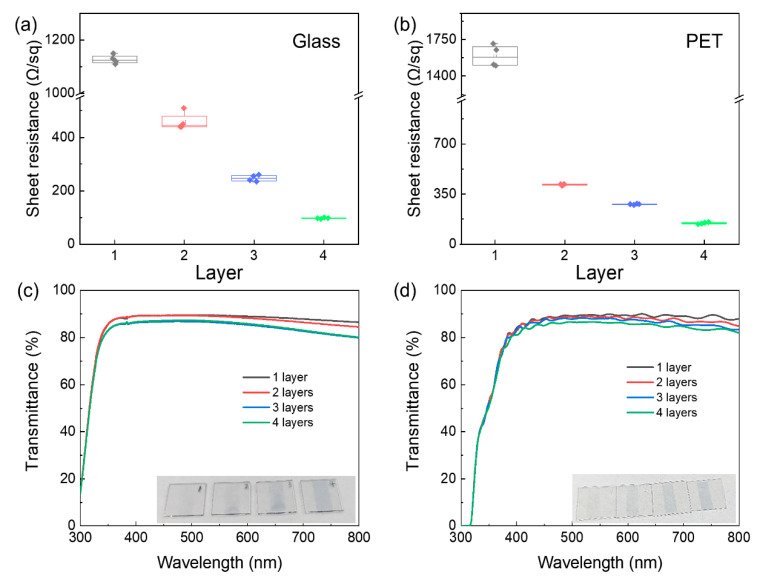
The sheet resistance dependent on the layer number of the PEDOT:PSS electrode on (**a**) glass and (**b**) PET substrates, respectively. The effect of layer number on the transmittance of the PEDOT:PSS electrode on (**c**) glass and (**d**) PET substrates, respectively. (Inset: images of PEDOT:PSS films with various layers on glass and PET substrates).

In order to evaluate the performance of a transparent electrode in the optoelectronic device, the conductivity and transparency of the inkjet-printed PEDOT:PSS electrode was studied. This work explored the influence of the substrate and layer number on the sheet resistance and transmittance of the PEDOT:PSS film. Figure 3a presented that the sheet resistance of PEDOT:PSS films decreased from 1127 Ω/sq for 1 layer to 460, 247, and 97 Ω/sq for 2, 3, and 4 layers, corresponding to the transmittance of 89.4% to 88.9%, 86.3% and 86.8% at 550 nm, respectively (Figure 3c). In contrast, Figure 3b demonstrated that the sheet resistance of the flexible PEDOT:PSS films decreased from 1592 Ω/sq for 1 layer to 416, 280, and 135 Ω/sq for 2, 3, and 4 layers, corresponding to the transmittance of 89.2% to 88.7%, 88.0% and 86.4% at 550 nm, respectively (Figure 3d). The flexible PEDOT:PSS electrode demonstrated a little higher sheet resistance than the rigid electrode, which was attribute to the higher surface roughness of the flexible PET substrate.

**Figure 4:**
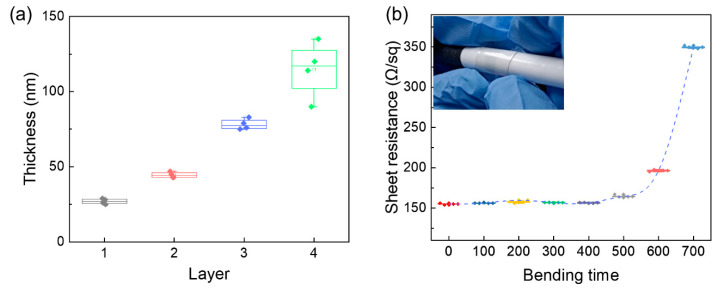
(**a**) The relationship between the film thickness and layer number. (**b**) The sheet resistance change of flexible PEDOT:PSS electrode dependent on the bending number. (Inset: images of bending test).

Moreover, the thicknesses of the printed PEDOT:PSS films were collected by a Surface Profilometer, which were 27 nm for 1 layer, 43 nm for 2 layers, 77 nm for 3 layers, and 110 nm for 4 layers. It can be observed from Figure 4a that the layer number and thickness are approximately linear-dependent, indicating that the thickness of PEDOT:PSS film was controllable by inkjet printing technology. Based on the sheet resistance and thickness, we calculated the conductivity of the printed PEDOT:PSS films. Notably, the four-layer conductivities of PEDOT:PSS film on glass and PET were 1213 and 871 S/cm, respectively. Meanwhile, a cylinder with a radius of 5 mm was used to conduct the bending test. The PET substrate printed with four-layer PEDOT:PSS electrode was repeatedly bent around the cylinder, and the sheet resistance was recorded after bending one hundred times. The bending test showed that the sheet resistance of the four-layer PEDOT:PSS electrode still retained 75% of its initial value after bending 600 times (Figure 4b). It proved that inkjet-printed PEDOT:PSS films on the PET substrate obtained an excellent mechanical flexibility. In summary, the four-layer PEDOT:PSS film on the PET substrate with a conductivity of 1213 S/cm, a sheet resistance of 97 Ω/sq and a transmittance of 86.8% possess the potential to act as a qualified OLED electrode.

**Figure 5:**
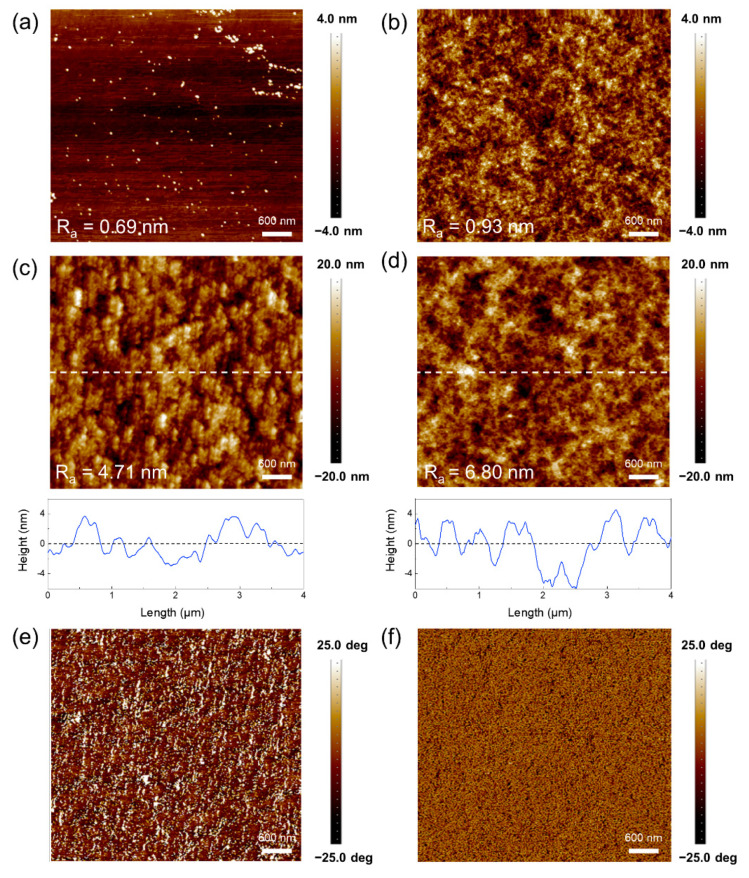
AFM surface morphology images of (**a**) glass and (**b**) PET substrates. AFM surface morphology images and surface lines of the inkjet-printed 4-layer PEDOT:PSS electrodes on (**c**) glass and (**d**) PET substrates. Phase image of the inkjet-printed 4-layer PEDOT:PSS electrodes on (**e**) glass and (**f**) PET substrates.

The morphology of PEDOT:PSS films on glass and PET substrates was observed via AFM. It can be observed from Figure 5a andb that the roughness of PET substrate (R_a_ = 0.93 nm) is larger than that of glass substrate (R_a_ = 0.69 nm), resulting in a higher roughness of PEDOT:PSS film on PET substrate. As shown in Figure 5c,d, the R_a_ of four-layer PEDOT:PSS are 4.71 nm and 6.80 nm on the glass and PET substrate, respectively. Notably, the R_a_ value of inkjet-printed four-layer PEDOT:PSS was higher than the conventional spin-coated films [13]. PEDOT and PSS domains can be distinguished in AFM phase image (Figure 5e,f), with dark areas corresponding to soft material PSS and bright areas corresponding to rigid material PEDOT. Additionally, it can be seen from the phase image that there are fibril-like phases in the printed PEDOT:PSS film, assigned to the crystalline PEDOT domain [35]. The fibril-like phase formation of PEDOT domains contributed the high conductivity [13,30].

**Figure 6:**
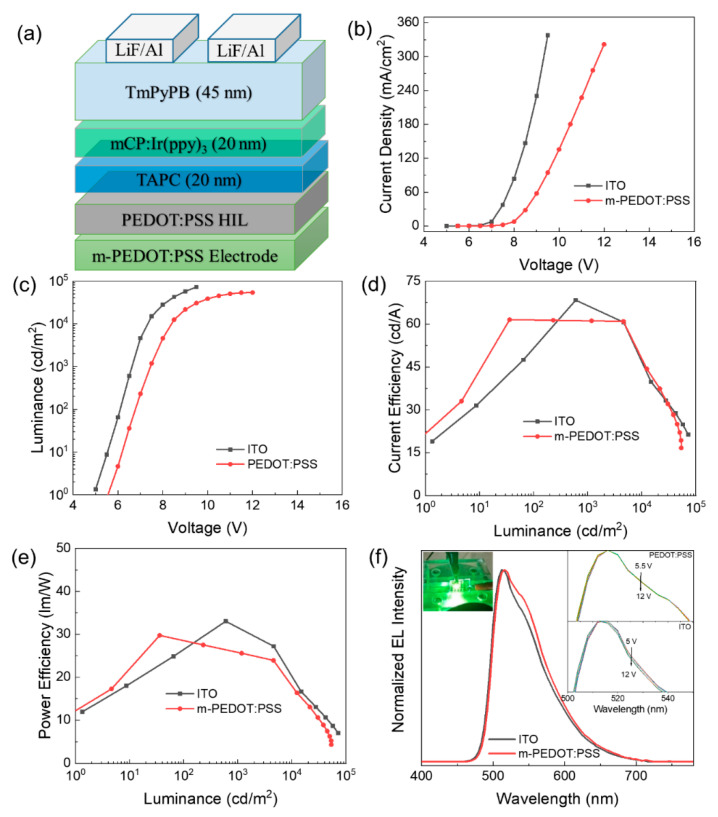
(**a**) Schematic device structure of the vacuum evaporated OLED. (**b**) Current density (*J*)-voltage (*V*), (**c**) luminance (*L*)-*V*, (**d**) current efficiency (CE)-*L*, (**e**) power efficiency (PE)-*L*, and (**f**) electroluminescence (EL) spectra of the OLEDs at the driving voltage of 9 V (Inset: The luminescence image and EL spectra at various driving voltages).

Based on the above discussion, the inkjet-printed four-layer PEDOT:PSS film meets the requirements of high conductivity, high transparency, and uniform film morphology for an electrode in optoelectronic devices. All of these characteristics make the inkjet-printed four-layer PEDOT:PSS film a promising anode for OLEDs. In this work, the inkjet-printed patterned four-layer PEDOT:PSS was used to fabricate green phosphorescence OLED with the device configuration as shown in Figure 6a, compared with the device with ITO as the anode. Figure 6b–f depicts the characteristics of current density (*J*)–voltage (*V*), luminance (*L*)–*V*, current efficiency (CE)–*L*, power efficiency (PE)–*L*, and electroluminescence (EL) spectra. The turn-on voltage, CE_max_ and PE_max_ were 5 V, 68 cd/A and 33 lm/W, respectively, for the ITO device, and 5.5 V, 61 cd/A and 29 lm/W, respectively, for the PEDOT:PSS device. We can see that the device performance of OLEDs with the inkjet-printed PEDOT:PSS and ITO electrodes are comparable in terms of CE and PE.

**Figure 7:**
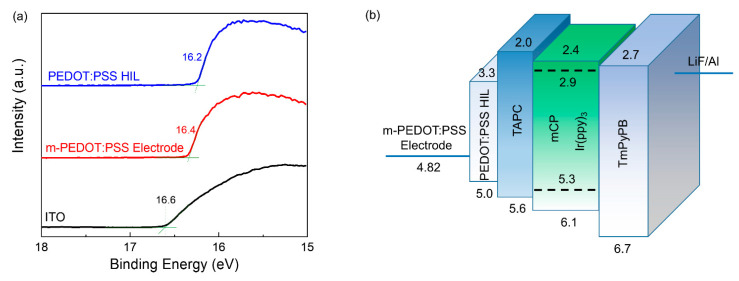
(**a**) The UPS spectra of ITO, PEDOT:PSS electrode, and PEDOT:PSS HIL. (**b**) The energy-level diagrams of the OLEDs.

Figure 7a shows the UPS spectra of ITO, PEDOT:PSS electrode, and PEDOT:PSS HIL. The work function can be determined by the difference between the incident photon energy (21.22 eV) and the binding energy of the secondary electron cut-off. Through theoretical calculation, the work functions are determined to be 4.62 eV, 4.82 eV, and 5.02 eV for ITO, PEDOT:PSS electrode and PEDOT:PSS HIL, respectively. Figure 7b shows the energy-level diagrams of the OLED. It was expected that the turn-on voltage of the PEDOT:PSS-anode should be lower than that of ITO-anode due to the decreased hole-injection barrier. However, in this work, the turn-on voltage of the PEDOT:PSS-anode device was 0.5 V higher and the current density was lower than that of the ITO-anode devices, which can be attributed to the relatively higher sheet resistance of the PEDOT:PSS electrode [36]. Notably, OLEDs with the PEDOT:PSS and ITO anodes obtained the same luminance at the same current density (Figure 6), resulting in the comparable device performance. Additionally, in the EL spectra of Figure 6f, the shoulder emission at 540 nm of the OLED with a PEDOT:PSS electrode was higher than the ITO device. Since Ir(ppy)_3_ is the only emissive component in the light-emitting layer, the small changes in the EL spectra are due to the cavity effect [37,38]. Here, the location of the electron–hole recombination zone within the light-emitting layer moved near the anode in the PEDOT:PSS device than in the ITO device, due to the higher sheet resistance. Furthermore, the PEDOT:PSS device performed better EL spectra stability than the ITO one at various driving voltages.

**Figure 8:**
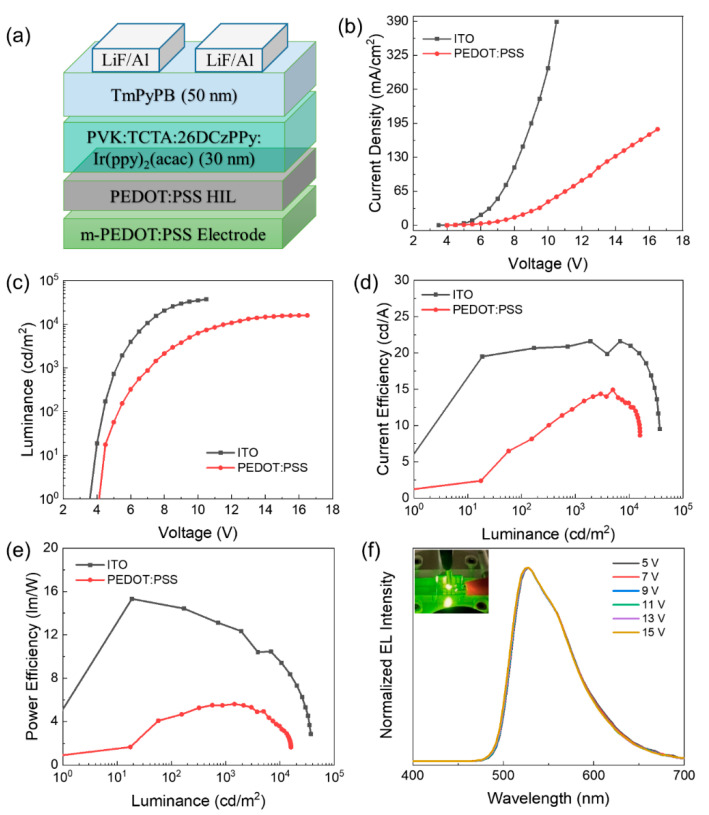
(**a**) Schematic device structure of the solution-processed OLED. (**b**) *J*-*V*, (**c**) *L*-*V*, (**d**) CE-*L*, (**e**) PE-*L*, and (**f**) EL spectra at different driving voltages of the OLEDs (Inset: The luminescence image).

Furthermore, all-solution process will be the mainstream technology for low-cost and large-area OLED displays in the near future. Therefore, the OLEDs with a spin-coated light-emitting layer were also fabricated in this work to explore the potential of the inkjet-printed PEDOT:PSS electrode in the full solution-processed OLEDs. The solution-processed OLEDs were fabricated with the device configuration as shown in Figure 8a. Figure 8b–f depicts the characteristics of (b) *J*-*V*, (c) *L*-*V*, (d) CE-*L,* (e) PE-*L*, and (f) EL spectra performance of the OLEDs. The turn-on voltage, CE_max_, PE_max_, and *L*_max_ for the solution-processed OLED were 3.5 V, 21.6 cd/A, 15.3 lm/W, and 37120 cd/m^2^, respectively for the ITO electrode and 4 V, 14.9 cd/A, 5.5 lm/W, and 15910 cd/m^2^, respectively for the PEDOT:PSS electrode. Compared with ITO devices, the efficiency of PEDOT:PSS electrode is obviously lower, due to the gap of the surface roughness and the conductivity between the two electrodes. Moreover, the device performance was inferior to the vacuum evaporation one, which can be attributed to the simple device structure, residue solvent, and lower packing density of the solution-processed emitting layer [39]. Thus, it is necessary to improve the solution-processed device performance by improving the film quality in our future research. Here, we demonstrated the potential application in highly efficient all solution-processed OLEDs in the future.

## 4. Conclusions

In conclusion, we added DMSO and Triton X-100 to the pristine PEDOT:PSS ink to improve the conductivity and spreadability on both of the rigid and flexible substrates. Moreover, EG and DI water were utilized to adjust the viscosity of the m-PEDOT:PSS ink, in order to enhance the printability and suppress the coffee-ring effect. Thus, the high transparent conductive PEDOT:PSS electrode was successfully fabricated with a conductivity of 1213 S/cm, a sheet resistance of 97 Ω/sq and a transmittance of 86.8% at 550 nm. In addition, the sheet resistance of flexible PEDOT:PSS electrode remained 75% of the original value after bending 600 times, demonstrating a good mechanical flexibility. Ultimately, the current efficiency of OLEDs prepared on the inkjet-printed PEDOT:PSS electrode was 61 cd/A, which was comparable to that of the ITO device. This work confirms the feasibility of inkjet printing technology to fabricate patterned electrodes and proves that inkjet printing technology can be used to fabricate highly efficient optoelectronic devices.

## Figures and Tables

**Table 1:** Boiling point, surface tension, and viscosity of the solvents and PEDOT:PSS inks with various formulations.

Inks	Boiling Point (°C)	Surface Tension (mN/m)	Viscosity (cP)
EG	197	48	20
DI water	100	72	1
m-PEDOT:PSS	-	-	44
m-PEDOT:PSS:DI water (2:1)	-	-	17
m-PEDOT:PSS:EG (1:1)	-	-	25
m-PEDOT:PSS:EG:DI water (2:2:1)	-	-	11

## References

[1] Aizawa N., Pu Y.J., Watanabe M., Chiba T., Ideta K., Toyota N., Igarashi M., Suzuri Y., Sasabe H., Kido J. (2014). Solution-processed multilayer small-molecule light-emitting devices with high-efficiency white-light emission. Nat. Commun..

[2] Han T.-H., Lee Y., Choi M.-R., Woo S.-H., Bae S.-H., Hong B.H., Ahn J.-H., Lee T.-W. (2012). Extremely efficient flexible organic light-emitting diodes with modified graphene anode. Nat. Photonics.

[3] Li N., Oida S., Tulevski G.S., Han S.J., Hannon J.B., Sadana D.K., Chen T.C. (2013). Efficient and bright organic light-emitting diodes on single-layer graphene electrodes. Nat. Commun..

[4] Ou Q.-D., Zhou L., Li Y.-Q., Shen S., Chen J.-D., Li C., Wang Q.-K., Lee S.-T., Tang J.-X. (2014). Extremely Efficient White Organic Light-Emitting Diodes for General Lighting. Adv. Funct. Mater..

[5] Wang Z.B., Helander M.G., Qiu J., Puzzo D.P., Greiner M.T., Hudson Z.M., Wang S., Liu Z.W., Lu Z.H. (2011). Unlocking the full potential of organic light-emitting diodes on flexible plastic. Nat. Photonics.

[6] Song W., Fanady B., Peng R., Hong L., Wu L., Zhang W., Yan T., Wu T., Chen S., Ge Z. (2020). Foldable Semitransparent Organic Solar Cells for Photovoltaic and Photosynthesis. Adv. Energy Mater..

[7] Chou W.-Y., Lin S.-T., Cheng H.-L., Chang M.-H., Guo H.-R., Wen T.-C., Mai Y.-S., Horng J.-B., Kuo C.-W., Tang F.-C. (2007). Polymer light-emitting diodes with thermal inkjet printed poly(3,4-ethylenedioxythiophene):polystyrenesulfonate as transparent anode. Thin Solid Films.

[8] Yoon S., Sohn S., Kwon J., Park J.A., Jung S. (2016). Double-shot inkjet printing for high-conductivity polymer electrode. Thin Solid Films.

[9] Wang T., Lu K., Xu Z., Lin Z., Ning H., Qiu T., Yang Z., Zheng H., Yao R., Peng J. (2021). Recent Developments in Flexible Transparent Electrode. Crystals.

[10] Du H., Guo Y., Cui D., Li S., Wang W., Liu Y., Yao Y., Zhao L., Dong X. (2021). Solution-processed PEDOT:PSS:GO/Ag NWs composite electrode for flexible organic light-emitting diodes. Spectrochim. Acta. A Mol. Biomol. Spectrosc..

[11] Lee J., Han T.H., Park M.H., Jung D.Y., Seo J., Seo H.K., Cho H., Kim E., Chung J., Choi S.Y. (2016). Synergetic electrode architecture for efficient graphene-based flexible organic light-emitting diodes. Nat. Commun..

[12] Tao F., Liu Y., Ren X., Jiang A., Wei H., Zhai X., Wang F., Stock H.-R., Wen S., Ren F. (2021). Carbon nanotube-based nanomaterials for high-performance sodium-ion batteries: Recent advances and perspectives. J. Alloys Compd..

[13] Liu L., Li S., Wu L., Chen D., Cao K., Duan Y., Chen S. (2021). Enhanced flexibility and stability of PEDOT:PSS electrodes through interfacial crosslinking for flexible organic light-emitting diodes. Org. Electron..

[14] Kommeren S., Coenen M.J.J., Eggenhuisen T.M., Slaats T.W.L., Gorter H., Groen P. (2018). Combining solvents and surfactants for inkjet printing PEDOT:PSS on P3HT/PCBM in organic solar cells. Org. Electron..

[15] Ely F., Avellaneda C.O., Paredez P., Nogueira V.C., Santos T.E.A., Mammana V.P., Molina C., Brug J., Gibson G., Zhao L. (2011). Patterning quality control of inkjet printed PEDOT:PSS films by wetting properties. Synth. Met..

[16] Darwis D., Sesa E., Elkington D., Sharafutdinova G., Lewis T., Zhou X., Dastoor P.C., Belcher W.J. (2021). Printing of PEDOT:PSS for top gate organic thin film transistor. J. Phys. Conf. Ser..

[17] Ahmed A., Jalil M.A., Hossain M.M., Moniruzzaman M., Adak B., Islam M.T., Parvez M.S., Mukhopadhyay S. (2020). A PEDOT:PSS and graphene-clad smart textile-based wearable electronic Joule heater with high thermal stability. J. Mater. Chem. C.

[18] Griffin J., Ryan A.J., Lidzey D.G. (2017). Solution modification of PEDOT:PSS inks for ultrasonic spray coating. Org. Electron..

[19] Singh A., Katiyar M., Garg A. (2015). Understanding the formation of PEDOT:PSS films by ink-jet printing for organic solar cell applications. RSC Adv..

[20] Ritruksa M., Wongrerkdee S., Lohawet K., Kaewprajak A., Kumnorkaew P., Wongrerkdee S. (2020). Surface modification of PEDOT: PSS film by chemical vapor texturing process for enhanced organic photovoltaics. Surf. Interfaces.

[21] Lin T., Sun X., Hu Y., Mu W., Sun Y., Zhang D., Su Z., Chu B., Cui Z. (2019). Blended host ink for solution processing high performance phosphorescent OLEDs. Sci. Rep..

[22] Derby B. (2010). Inkjet Printing of Functional and Structural Materials: Fluid Property Requirements, Feature Stability, and Resolution. Annu. Rev. Mater. Res..

[23] Tait J.G., Witkowska E., Hirade M., Ke T.-H., Malinowski P.E., Steudel S., Adachi C., Heremans P. (2015). Uniform Aerosol Jet printed polymer lines with 30μm width for 140ppi resolution RGB organic light emitting diodes. Org. Electron..

[24] Zhu H., Shin E.S., Liu A., Ji D., Xu Y., Noh Y.Y. (2019). Printable Semiconductors for Backplane TFTs of Flexible OLED Displays. Adv. Funct. Mater..

[25] McKinley G.H., Renardy M. (2011). Wolfgang von Ohnesorge. Phys. Fluids.

[26] Krainer S., Smit C., Hirn U. (2019). The effect of viscosity and surface tension on inkjet printed picoliter dots. RSC Adv..

[27] Shin P., Sung J., Lee M.H. (2011). Control of droplet formation for low viscosity fluid by double waveforms applied to a piezoelectric inkjet nozzle. Microelectron. Reliab..

[28] Du Z., Zhou H., Yu X., Han Y. (2020). Controlling the polarity and viscosity of small molecule ink to suppress the contact line receding and coffee ring effect during inkjet printing. Colloids Surf. A Physicochem. Eng. Asp..

[29] Amruth C., Szymanski M.Z., Luszczynska B., Ulanski J. (2019). Inkjet Printing of Super Yellow: Ink Formulation, Film Optimization, OLEDs Fabrication, and Transient Electroluminescence. Sci. Rep..

[30] Teo M.Y., RaviChandran N., Kim N., Kee S., Stuart L., Aw K.C., Stringer J. (2019). Direct Patterning of Highly Conductive PEDOT:PSS/Ionic Liquid Hydrogel via Microreactive Inkjet Printing. ACS Appl. Mater. Interfaces.

[31] Mu L., Hu Z., Zhong Z., Jiang C., Wang J., Peng J., Cao Y. (2017). Inkjet-printing line film with varied droplet-spacing. Org. Electron..

[32] Basak I., Nowicki G., Ruttens B., Desta D., Prooth J., Jose M., Nagels S., Boyen H.G., D’Haen J., Buntinx M. (2020). Inkjet Printing of PEDOT:PSS Based Conductive Patterns for 3D Forming Applications. Polymers.

[33] Eom S.H., Senthilarasu S., Uthirakumar P., Yoon S.C., Lim J., Lee C., Lim H.S., Lee J., Lee S.-H. (2009). Polymer solar cells based on inkjet-printed PEDOT:PSS layer. Org. Electron..

[34] Sleczkowski P., Borkowski M., Zajaczkowska H., Ulanski J., Pisula W., Marszalek T. (2020). Geometry Control of Source/Drain Electrodes in Organic Field-Effect Transistors by Electrohydrodynamic Inkjet Printing. Materials.

[35] Lo L.W., Zhao J., Wan H., Wang Y., Chakrabartty S., Wang C. (2021). An Inkjet-Printed PEDOT:PSS-Based Stretchable Conductor for Wearable Health Monitoring Device Applications. ACS Appl. Mater. Interfaces.

[36] Cai M., Ye Z., Xiao T., Liu R., Chen Y., Mayer R.W., Biswas R., Ho K.M., Shinar R., Shinar J. (2012). Extremely efficient indium-tin-oxide-free green phosphorescent organic light-emitting diodes. Adv. Mater..

[37] Meerheim R., Scholz S., Olthof S., Schwartz G., Reineke S., Walzer K., Leo K. (2008). Influence of charge balance and exciton distribution on efficiency and lifetime of phosphorescent organic light-emitting devices. J. Appl. Phys..

[38] So F., Krummacher B., Mathai M.K., Poplavskyy D., Choulis S.A., Choong V.-E. (2007). Recent progress in solution processable organic light emitting devices. J. Appl. Phys..

[39] Lee T.-W., Noh T., Shin H.-W., Kwon O., Park J.-J., Choi B.-K., Kim M.-S., Shin D.W., Kim Y.-R. (2009). Characteristics of Solution-Processed Small-Molecule Organic Films and Light-Emitting Diodes Compared with their Vacuum-Deposited Counterparts. Adv. Funct. Mater..

